# Determinants of Efficacy and Optimization of Chimeric Antigen Receptor T-Cell Therapy for Treating Multiple Myeloma: Current Status and Future Perspectives

**DOI:** 10.3390/cells15040380

**Published:** 2026-02-23

**Authors:** Hiroshi Yasui, Noriko Doki, Wei Yan, Kohzoh Imai, Tadao Ishida

**Affiliations:** 1 Department of Hematology and Oncology, St. Marianna University School of Medicine, Kawasaki 216-8511, Japan; 2 The Institute of Medical Science, The University of Tokyo, Tokyo 108-8639, Japan; 3 Hematology Division, Tokyo Metropolitan Cancer and Infectious Diseases Center, Komagome Hospital, Tokyo 113-8677, Japan; noriko_doki@tmhp.jp; 4 Department of Hematology, Shengjing Hospital of China Medical University, Shenyang 110004, China; helen809010@163.com; 5 Sapporo Medical University, Sapporo 060-8556, Japan; kima@ims.u-tokyo.ac.jp; 6 Institute for Genetic Medicine, Hokkaido University, Sapporo 060-0808, Japan; 7 Kanagawa Cancer Center Research Institute, Yokohama 241-8515, Japan; 8 Department of Hematology, Japanese Red Cross Medical Center, Tokyo 150-8935, Japan; i.s.h.i.28@rondo.ocn.ne.jp

**Keywords:** chimeric antigen receptor T-cell therapy, multiple myeloma, B-cell maturation antigen, tumor burden, soluble B-cell maturation antigen, bridging therapy, biomarkers, resistance

## Abstract

**Highlights:**

**What are the main findings?**

- Chimeric antigen receptor (CAR) T-cell outcomes in multiple myeloma (MM) are shaped by the biological context of the infusion delivered. This can include tumor burden, antigen dynamics, and the functional fitness of the cells themselves.
- Effector-to-target balance and soluble B-cell maturation antigen also serve as actionable biomarkers that link disease burden to efficacy and safety.

**What are the implications of the main findings?**

- The optimization of CAR T-cell therapy requires biomarker-guided patient selection, treatment timing, and pre-infusion disease control beyond CAR construct design alone.
- A design-oriented, patient-centered framework may improve the durability and consistency of clinical benefits experienced from this therapy by patients with MM.

**Abstract:**

Chimeric antigen receptor (CAR) T-cell therapy has transformed the treatment of relapsed and refractory multiple myeloma (MM), with BCMA-directed products demonstrating unprecedented response rates in heavily pretreated patients. Despite these advances, variabilities in response durability, treatment-related toxicities, and the emergence of resistance underscore the need for strategies that extend beyond CAR construct design alone. Accumulating evidence has indicated that the therapeutic outcomes of this approach are determined by a complex interplay between tumor burden, antigen dynamics, CAR T-cell functional fitness, and host immune context at the time of infusion. Effector-to-target balance and antigen load, in particular, have emerged as modifiable biological determinants of efficacy and safety, with pre-infusion disease control and response to bridging therapy exerting a profound influence on post-infusion CAR T-cell expansion, persistence, and clinical outcomes. Soluble BCMA (sBCMA) has also gained increasing attention as a practical biomarker that integrates tumor burden and antigen dynamics to facilitate the biologically informed optimization of treatment timing and patient selection. In addition to tumor- and antigen-related factors, the intrinsic properties of CAR T-cell products—including the spatial organization and clustering of CAR molecules on the T-cell surface—represent an additional layer of biological determinants that correlate with treatment responses. The quantitative functional assessment of CAR T-cell products may complement conventional clinical and tumor-based biomarkers and improve the prediction of therapeutic potency prior to infusion. This review summarizes recent advances in CAR T-cell therapy for treating MM, focusing on key mechanisms of resistance, the optimization of pre-infusion disease control, the integration of biological markers into clinical decision-making, and emerging combinations and sequential strategies. We also propose a design-oriented and patient-centered framework that integrates CAR engineering with disease biology and host immune factors to enhance the consistency, durability, and safety of CAR T-cell therapy. Such biologically guided optimization strategies will likely prove critical for fully realizing the transformative potential of CAR T-cell therapy across the evolving treatment continuum of MM.

## 1. Introduction

Multiple myeloma (MM) remains a largely incurable plasma cell malignancy, despite substantial advances in therapeutic options over the past two decades. The introduction of proteasome inhibitors, immunomodulatory drugs, and monoclonal antibodies has significantly improved patient outcomes. However, most patients eventually develop relapsed or refractory disease. In this context, chimeric antigen receptor (CAR) T-cell therapy has emerged as a transformative immunotherapeutic approach that has offered unprecedented response rates and robust remission states in heavily pretreated patients. In particular, CAR T-cell therapies that target the B-cell maturation antigen (BCMA) have demonstrated promising clinical efficacy and have been rapidly incorporated into the treatment landscape for MM, fundamentally changing current expectations for MM control in advanced settings.

Despite these successes, accumulated clinical evidence has also revealed certain key limitations of CAR T-cell therapy for MM. A substantial proportion of patients who receive this treatment relapse after initially achieving a robust response, and the treatment-related toxicities—including cytokine release syndrome and neurotoxicity—remain clinically significant. Moreover, the durability of the response varies widely among patients, highlighting a significant heterogeneity in treatment outcomes. These observations suggest that the efficacy of CAR T-cell therapy cannot be explained solely by target antigen expression or CAR construct design. Rather, emerging evidence indicates that clinical outcomes are shaped by a complex interplay of factors that operate before and after CAR T-cell infusion. These include the disease burden at the time of treatment, the effectiveness of bridging therapy, intrinsic T-cell fitness, and dynamic interactions between the tumor and immune system. Understanding these determinants is essential for optimizing patient selection, treatment timing, and next-generation CAR T-cell strategies for MM.

**Figure 1:**
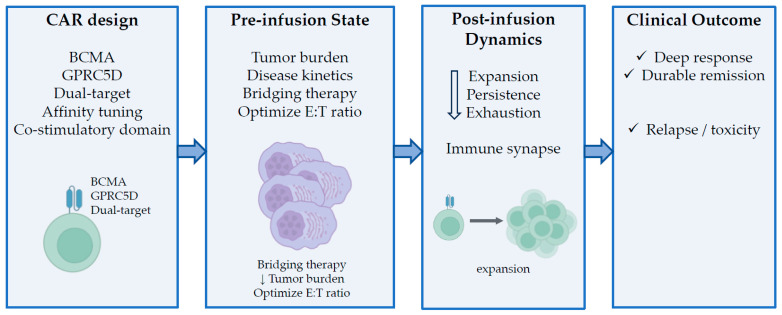
Conceptual framework of the determinants of efficacy and optimization strategies for CAR T-cell therapy for treating multiple myeloma. This figure illustrates an integrated, design-oriented framework that highlights the key determinants of efficacy and strategies for optimizing CAR T-cell therapy in MM. CAR construct features and target selection, including BCMA, GPRC5D, and dual-target approaches, interact with the pre-infusion disease state, which is characterized by tumor burden, disease kinetics, and the effects of bridging therapy. These factors collectively influence the E:T balance at the time of infusion. Post-infusion CAR T-cell dynamics, including expansion, persistence, exhaustion, and immune synapse formation, further shape therapeutic outcomes. Biological correlates of responses, such as the E:T ratio, antigen dynamics, and product-level functional characteristics, provide mechanistic insights into these different phases. Clinical outcomes (e.g., robust and durable responses, relapse, or toxicity) emerge from the interplay of these multidimensional factors, underscoring the notion that CAR T-cell efficacy is determined by processes spanning both pre- and post-infusion stages.

This review aims to move beyond a descriptive summary of clinical trials, providing an integrated and concept-driven analysis of CAR T-cell therapy for MM. First, we outline the current clinical landscape of BCMA-directed CAR T-cell therapies, highlighting their achievements and limitations in real-world clinical practice. We then systematically examine the key determinants of therapeutic efficacy, focusing on disease burden at the time of infusion, the role of bridging therapy, intrinsic T-cell fitness, and various emerging biological correlates of response. We then discuss recent advances in target antigen selection—including G protein-coupled receptor class C group 5 member D (GPRC5D), dual-target CAR strategies, and the structural optimization of CAR constructs. By synthesizing clinical and biological insights across these domains, this review seeks to establish a conceptual framework for understanding responses and resistance to CAR T-cell therapy in the context of MM, as well as propose rational strategies for optimizing treatment outcomes and guiding the development of next-generation CAR T-cell therapies. This review discusses the key determinants of CAR T-cell efficacy and failure in the treatment of MM, and proposes an integrated, design-oriented framework spanning CAR construct features, pre-infusion disease state, biological correlates, and clinical outcomes (Figure 1).

## 2. Current Landscape of CAR T-Cell Therapy for MM

The current clinical landscape of CAR T-cell therapy for MM is largely defined by the development and clinical implementation of BCMA-directed approaches. BCMA is selectively expressed on the surfaces of malignant plasma cells and plays a critical role in the survival of myeloma cells. This makes it an attractive therapeutic target for treating MM [1]. Early-phase clinical trials of BCMA-targeted CAR T-cell therapies have demonstrated unprecedented overall response rates and robust responses in heavily pretreated patients with relapsed or refractory MM [2], rapidly establishing CAR T-cell therapy as a highly effective treatment modality for advanced forms of the disease. These promising results have since been confirmed and extended in larger studies and real-world clinical experience, leading to regulatory approval and the broader clinical adoption of BCMA-directed CAR T-cell therapies that have firmly positioned them as a central component of modern MM treatment strategies.

### 2.1. BCMA-Targeted CAR T-Cell Therapies

Among the various BCMA-directed CAR T-cell therapies, idecabtagene vicleucel (ide-cel) and ciltacabtagene autoleucel (cilta-cel), represent the most clinically advanced and widely adopted platforms that have set the current standard for this form of MM treatment. Both represent autologous CAR T-cell therapies that target BCMA; however, they differ in their CAR construct design, including their antigen-binding domains and signaling architecture, which may contribute to their differences in clinical activity and response durability. Ide-cel was the first BCMA-directed CAR T-cell therapy to demonstrate robust efficacy and an acceptable safety profile in heavily pretreated patients, establishing a proof of concept for BCMA-targeting in MM. Building on this foundation, cilta-cel incorporates a distinct CAR design with dual BCMA-binding domains. This drug has been associated with more robust and durable responses in clinical trials. Together, ide-cel and cilta-cel exemplify the rapid evolution of BCMA-targeted CAR T-cell therapy and provide a framework for understanding how CAR design and biological factors can influence clinical MM outcomes.

A summary of clinically advanced BCMA-directed CAR T-cell therapies and their key clinical outcomes is provided in Table 1. Pivotal clinical trials of ide-cel [2,3] and cilta-cel [4,5] have consistently demonstrated high overall response rates and a substantial proportion of robust responses, including complete and stringent complete responses, in patients with heavily pretreated, relapsed, or refractory MM. These responses have translated into meaningful improvements in progression-free survival compared with historical controls, confirming the transformative potential of BCMA-targeted CAR T-cell therapy. Nevertheless, follow-up data have revealed considerable interpatient variability in the robustness and durability of response, with a subset of patients experiencing early relapse despite initial tumor clearance. Real-world studies have largely corroborated the efficacy observed in clinical trials, while also highlighting practical challenges such as manufacturing time, patient selection, disease progression during bridging therapy, and the management of treatment-related toxicities [6]. Together, these observations underscore the notion that, although BCMA-directed CAR T-cell therapies have established a new benchmark for efficacy in treating advanced MM, their clinical outcomes are influenced by factors that extend beyond the CAR construct itself. This has set the stage for a deeper examination of the determinants of response and resistance.

### 2.2. Limitations of First-Generation BCMA CAR T-Cell Therapy

Despite the high clinical efficacy achieved by first-generation BCMA-targeted CAR T-cell therapies, accumulating clinical experience has revealed several intrinsic limitations that constrain their long-term therapeutic effects on MM. Relapse after an initial robust response remains a common clinical challenge, underscoring the limited durability of disease control in a substantial proportion of patients. Treatment-related toxicities—including cytokine release syndrome and immune effector cell-associated neurotoxicity syndrome—continue to require specialized management, limiting the broader applicability of this therapy in vulnerable patient populations. Beyond these immediate concerns, the heterogeneity observed in clinical outcomes between patients with seemingly similar disease characteristics highlights the notion that MM responses to BCMA-directed CAR T-cell therapy are not uniform. These limitations suggest that factors such as disease burden at infusion, pretreatment disease control, T-cell quality, and dynamic tumor–immune interactions play critical roles in terms of shaping therapeutic outcomes, thereby requiring a more nuanced understanding of the determinants of efficacy beyond target antigen expression alone.

## 3. Determinants of CAR T-Cell Efficacy in MM

Clinical outcomes following CAR T-cell therapy for multiple myeloma are not determined by CAR construct design alone but arise from the interaction of several biological determinants operating before and after infusion. These determinants include disease burden and disease kinetics at the time of infusion, and the functional fitness of infused T cells, all of which have been implicated in shaping in vivo CAR T-cell expansion, persistence, and clinical durability [2]. A structured understanding of these determinants is essential for interpreting heterogeneous clinical outcomes and for optimizing CAR T-cell therapy in MM.

### 3.1. Tumor Burden and Disease Status at the Time of CAR T-Cell Infusion

Tumor burden and disease status at the time of CAR T-cell infusion have emerged as critical determinants of its therapeutic efficacy for MM. Clinical studies and real-world analyses have consistently demonstrated that patients with lower disease burdens at the time of infusion achieve deeper and more durable responses than those with high tumor loads or rapidly progressing MM [2,7,8]. A high baseline tumor burden is associated with an unfavorable E:T balance, which can limit effective CAR T-cell expansion and promote early functional exhaustion. Aggressive disease biology—characterized by high-risk cytogenetic features, extramedullary disease, or rapid disease kinetics—can further compromise CAR T-cell activity by overwhelming immune-mediated tumor control mechanisms [9].

The impact of disease status at infusion extends beyond initial response rates to influence the durability of remission. Patients who achieve substantial cytoreduction prior to receiving CAR T-cell infusion are more likely to sustain prolonged progression-free survival, whereas those who have uncontrolled MM at infusion frequently experience early relapse despite initial tumor clearance. These observations suggest that tumor burden is not merely a surrogate marker of disease severity, but also an active biological determinant that shapes CAR T-cell dynamics in vivo. Consequently, the assessment of a patient’s disease burden at the time of initial infusion—including tumor volume, circulating tumor markers, and disease kinetics—should be regarded as a central component of patient selection and treatment planning for CAR T-cell therapy in the treatment of MM.

### 3.2. Impact of Bridging Therapy

Given the manufacturing timelines required for CAR T-cell production, as well as the aggressive disease course frequently observed in heavily pretreated MM, bridging therapy has become an integral component of CAR T-cell treatment strategies. In pivotal clinical trials of ide-cel and cilta-cel, substantial proportions of patients required bridging therapy between leukapheresis and CAR T-cell infusion [2]. In a large multicenter real-world analysis of patients receiving standard-of-care ide-cel in the United States, bridging therapy was administered in approximately 80% of patients and consisted mainly of steroid-based or combination regimens, including proteasome inhibitor-based combinations, IMiD ± monoclonal antibody-based regimens, alkylator-based therapy (most commonly cyclophosphamide), selinexor-containing regimens, and, in selected cases, focal radiotherapy. High-intensity multi-agent chemotherapy was used infrequently due to prolonged myelosuppression and infection risk [10].

Recent post hoc analyses of the phase 3 CARTITUDE-4 trial have provided the most robust evidence to date supporting the clinical relevance of effective bridging therapy [4,11]. In that study, patients who received cilta-cel and achieved a ≥25% reduction in tumor burden during bridging therapy demonstrated significantly longer progression-free survival times than those with lesser degrees of disease control [11]. These findings establish the response to bridging therapy as a clinically meaningful determinant of CAR T-cell efficacy, rather than merely a logistical consideration. Importantly, mechanistic analyses from the CARTITUDE-4 trial have further linked effective bridging therapy to favorable biological correlates at the time of infusion. Patients with greater tumor burden reduction exhibited lower pre-infusion sBCMA levels and significantly higher in vivo effector-to-target (E:T) ratios, primarily driven by reduced tumor burden.

### 3.3. Biological Correlates of Responses to CAR T-Cell Therapy

Beyond clinical variables such as tumor burden and disease kinetics, increasing evidence indicates that CAR T-cell efficacy in MM is governed by a set of biological correlates that link pre-infusion disease status to post-infusion cellular behavior [7]. These correlates provide mechanistic insights into why patients with similar clinical characteristics may experience markedly different outcomes following CAR T-cell therapy.

Among these, the E:T ratio at the time of infusion has emerged as a major determinant of response. A higher E:T balance, reflecting a favorable relationship between functional CAR T-cells and antigen-bearing tumor cells, has been associated with more favorable CAR T-cell expansion and progression-free survival [11]. Notably, recent analyses have suggested that this balance is more strongly influenced by tumor burden than by absolute CAR T-cell dose or peak expansion alone.

An additional biologically relevant factor is BCMA shedding, which contributes to elevated levels of soluble BCMA (sBCMA) and can reduce effective CAR–antigen engagement by acting as a decoy. Early consideration of BCMA shedding is important, as it links tumor burden, soluble antigen dynamics, and resistance mechanisms observed after CAR T-cell infusion. Accordingly, sBCMA represents a complementary biomarker that integrates tumor burden with antigen dynamics and can serve as a surrogate measure of overall antigen load.

## 4. Evolution of Target Antigens and CAR Design

Although BCMA-directed CAR T-cell therapies have established a new standard of efficacy in MM, the limitations observed in terms of response durability and relapse patterns have underscored the need to expand therapeutic strategies beyond single-antigen targeting [2]. Clinical relapses after BCMA CAR T-cell therapy have been frequently associated with antigen downregulation, heterogeneous target expression, or the selective outgrowth of antigen-negative tumor clones. This highlights the vulnerabilities inherent in first-generation CAR T-cell approaches. These challenges have prompted intensive efforts to identify alternative target antigens, refine the CAR design to overcome resistance mechanisms, and extend the therapeutic benefits of CAR T-cell therapy in MM.

Recent advances in target discovery and CAR engineering have broadened the antigenic landscape of CAR T-cell therapy beyond BCMA alone [12]. Novel targets such as GPRC5D, as well as dual-target and multispecific CAR strategies, have emerged as promising approaches to address antigen escape and enhance treatment durability. In parallel, the structural optimization of CAR constructs, including modifications to antigen-binding domains, costimulatory signaling, and spacer regions, has aimed to improve T-cell persistence, functional fitness, and safety. Together, these developments represent an evolution from first-generation BCMA-focused therapies toward more sophisticated and resilient CAR T-cell platforms, reflecting a deeper understanding of tumor heterogeneity and tumor–immune system dynamics in MM.

### 4.1. GPRC5D CAR T-Cell Therapies

GPRC5D has emerged as one of the most promising alternative target antigens for CAR T-cell therapy in the context of MM [13]. This protein is highly expressed on the surfaces of malignant plasma cells. Conversely, it shows very limited expression in normal tissues (with the notable exception of keratinized structures), making it an attractive target in terms of both efficacy and safety. GPRC5D expression appears to be largely independent of BCMA, providing a biologically distinct antigenic axis that can be exploited to overcome the resistance associated with BCMA downregulation or loss.

Early-phase clinical studies of GPRC5D-directed CAR T-cell therapies have demonstrated encouraging antimyeloma activity, including high response rates in patients with relapsed or refractory disease who have previously received BCMA-targeted therapies [14]. These findings highlight the potential role of GPRC5D CAR T-cells as an effective salvage strategy in the event of BCMA CAR T-cell therapy failure. Notably, clinical responses have been observed even in patients who previously underwent multiple lines of therapy, reinforcing the notion that antigen switching can restore CAR T-cell sensitivity in the context of antigen escape [15].

From a mechanistic perspective, targeting GPRC5D addresses a key limitation of first-generation BCMA-directed approaches by reducing dependence on a single antigenic pathway. However, GPRC5D CAR T-cell therapy carries certain inherent challenges as well. On-target, off-tumor toxicities related to GPRC5D expression in keratinized tissues such as the skin and nails have been reported, requiring careful monitoring and management. The long-term durability of responses and resistance patterns following GPRC5D-directed therapy also remain to be fully elucidated [16]. Collectively, these observations suggest that GPRC5D-based CAR T-cell therapies represent a critical component of next-generation CAR T-cell strategies for treating MM—particularly after BCMA approaches have failed or lost durability, underscoring the need for continued optimization regarding target selection and CAR design.

### 4.2. Dual-Target and Bispecific CAR T-Cell Approaches

Dual-target and bispecific CAR T-cell approaches have emerged as rational strategies to address antigen heterogeneity and escape that are being increasingly recognized as major mechanisms underlying relapse after single-antigen CAR T-cell therapy for MM. By concurrently targeting two distinct antigens—most commonly BCMA and GPRC5D—these approaches aim to reduce selective pressure on any single antigenic axis, thus enhancing the robustness of tumor control [17]. This strategy is conceptually supported by clinical observations that have demonstrated heterogeneous antigen expression within and across myeloma clones, particularly following BCMA treatment.

Early clinical studies evaluating dual-target CAR T-cell therapies—including constructs that incorporate two antigen-binding domains within a single CAR, or strategies involving the co-administration of distinct CAR T-cell products—have shown promising antimyeloma activity [18]. In particular, the dual targeting of BCMA and GPRC5D has shown high response rates in heavily pretreated patients, including those with prior exposure to BCMA-directed therapies [18]. These findings suggest that the concurrent engagement of multiple tumor-associated antigens may mitigate the risk of antigen-negative relapse and enhance response robustness by broadening immune pressure across heterogeneous tumor populations.

Bispecific CAR T-cell designs further expand this concept by facilitating flexible antigen recognition and potentially modulating the intensity of CAR signaling depending on antigen availability. Such designs may allow CAR-T cells to maintain their activity even when the expression of one target antigen is reduced, thereby preserving effector function even in the context of dynamic tumor evolution. However, dual-target and bispecific approaches also introduce new challenges such as the increased complexity of CAR design, potential additive toxicities, and the need to balance signaling strength to avoid excessive activation or exhaustion. Ongoing clinical and translational studies are therefore essential to define the optimal configurations, sequencing, and clinical contexts in which these next-generation CAR T-cell strategies can deliver durable and safe therapeutic benefits to patients with MM.

### 4.3. Structural Optimization of CAR Constructs

In parallel with the expansion of target antigen strategies, the structural optimization of CAR constructs has emerged as a critical approach to improving the efficacy, durability, and safety of CAR T-cell therapy for MM. CAR architecture—including antigen-binding domains, hinge and spacer regions, transmembrane domains, and intracellular signaling motifs—plays a central role in shaping T-cell activation, persistence, and exhaustion [19].

The optimization of antigen-binding domains has focused on tuning affinity and epitope specificity to balance effective tumor recognition while avoiding the excessive tonic signaling that can drive early T-cell exhaustion. Hinge and spacer lengths also influence antigen accessibility and immune synapse formation, thereby modulating activation thresholds. These factors are particularly relevant in MM, where antigen density and spatial distribution can vary across disease stages [20].

Intracellular signaling domains represent another key determinant of CAR T-cell behavior. The choice of costimulatory domains such as CD28 or 4-1BB differentially affects expansion kinetics, metabolic programming, and persistence, with CD28-based CARs favoring rapid expansion while 4-1BB-based designs promote enhanced longevity [20,21]. Emerging constructs incorporating alternative or combinatorial signaling motifs aim to optimize both early efficacy and long-term disease control.

Beyond these established design elements, recent studies have begun to highlight the importance of the structural and spatial features of CAR expression that are not captured by conventional construct-level descriptors. Quantitative assessments of CAR molecule localization and clustering on T-cell surfaces have shown that these properties reflect immune synapse formation and cytotoxic potential. Advanced imaging flow cytometry-based analyses have further revealed that differences in the molecular distribution of CAR correlate with functional activity and antitumor efficacy, suggesting that product-level spatial organization represents an additional and previously underappreciated aspect of CAR T-cell optimization [22].

### 4.4. Allogeneic CAR T-Cell Therapy

Allogeneic CAR T-cell therapy has emerged as a promising next-generation approach aimed at overcoming several limitations of autologous CAR T-cell products, including manufacturing delays, variable product quality, and limited accessibility [23]. The use of healthy donor-derived T cells enable the development of off-the-shelf CAR T-cell products with more consistent functional fitness and rapid availability, which may be particularly advantageous for patients with aggressive or rapidly progressive MM. Early-phase clinical studies evaluating allogeneic BCMA-directed CAR T-cell therapies have demonstrated preliminary antimyeloma activity; however, several challenges remain [24]. These include limited in vivo persistence due to host immune rejection, the need for genetic modifications to prevent graft-versus-host disease, and the requirement for additional lymphodepleting or immunosuppressive strategies [25,26]. As a result, the durability of responses observed to date has generally been inferior to that achieved with autologous CAR T-cell therapy. Despite these challenges, ongoing advances in gene-editing technologies and immune evasion strategies may improve the safety, persistence, and clinical efficacy of allogeneic CAR T-cell platforms. Continued clinical evaluation will be required to define the optimal role of allogeneic CAR T-cell therapy within the evolving treatment landscape of MM.

### 4.5. Comparison of CAR T-Cell Therapy and CD38-Targeted Antibody-Based Therapies

CAR T-cell therapy and CD38-targeted antibody-based therapies represent two major immunotherapeutic modalities in the treatment of MM, each with distinct mechanisms of action and clinical roles. CD38-targeted monoclonal antibodies, such as daratumumab and isatuximab, exert antimyeloma activity through immune-mediated mechanisms including antibody-dependent cellular cytotoxicity, complement-dependent cytotoxicity, and immune modulation, and have become integral components of both frontline and relapsed treatment regimens [27,28].

In contrast, CAR T-cell therapy is characterized by the capacity to induce deep and often rapid tumor reduction through direct cytotoxic engagement, resulting in high rates of minimal residual disease negativity and durable responses in heavily pretreated patients [2]. However, CAR T-cell therapy is associated with unique challenges, including manufacturing time, limited accessibility, and treatment-related toxicities such as cytokine release syndrome and neurotoxicity. Rather than representing competing strategies, CAR T-cell therapy and CD38-targeted antibody-based therapies occupy complementary positions within the therapeutic landscape of MM. CD38-targeted antibodies provide broad applicability and sustained disease control across treatment lines, whereas CAR T-cell therapy offers the potential for profound disease debulking and prolonged remission in selected patients. Optimal treatment sequencing and combination strategies will be essential to maximize the benefits of these distinct immunotherapeutic approaches.

## 5. Mechanisms of Resistance and Failure

Despite substantial advances in target selection and CAR construct design, resistance and treatment failure remain significant challenges in CAR T-cell therapy for MM. Clinical relapses after CAR T-cell therapy vary in their timing and presentation, reflecting the multifactorial nature of resistance mechanisms. These mechanisms can be broadly categorized into antigen-related escape, intrinsic CAR T-cell dysfunction, and host- or microenvironment-mediated resistance. These features are often co-present and interact during disease progression.

### 5.1. Antigen-Related Escape Mechanisms

The loss or modulation of target antigen expression represents one of the most well-characterized mechanisms of MM resistance following CAR T-cell therapy [29]. In the context of BCMA-directed CAR T-cell therapy, disease relapse has been associated with reduced surface expression of BCMA, altered antigen processing, and the selection of antigen-negative tumor clones [30,31]. The shedding of BCMA, which raises sBCMA levels, can further impair CAR T-cell engagement by decreasing the effective antigen density or acting as a decoy [32]. Collectively, these antigen-related escape mechanisms underscore the inherent vulnerability of strategies that target only single antigens and provide a biological rationale for the alternative and dual-target approaches discussed in Section 4 [12].

### 5.2. Intrinsic CAR T-Cell Dysfunction

Intrinsic CAR T-cell dysfunction is a major contributor to treatment failure following CAR T-cell therapy. Early T-cell exhaustion, impaired proliferative capacity, and suboptimal persistence can limit sustained antitumor activity, even in the presence of adequate target antigen expression. CAR design features—including tonic signaling induced by high-affinity antigen binding or specific costimulatory domains—can further predispose CAR T-cells to functional exhaustion [21,33]. The quality of autologous T cells at the time of manufacturing, which is heavily influenced by prior therapies, disease burden, and patient-related factors, profoundly influences CAR T-cell fitness and long-term efficacy [34].

### 5.3. Host and Tumor Microenvironment-Mediated Resistance

The host immune context and tumor microenvironment further modulate CAR T-cell activity and contribute to resistance [35]. Immunosuppressive cellular components, inhibitory cytokines, and metabolic constraints within the bone marrow niche can impair CAR T-cell function after infusion [35,36,37]. Inflammatory responses associated with treatment-related toxicities can paradoxically accelerate T-cell dysfunction or limit dose intensity [38]. These host- and microenvironment-mediated factors highlight the complexity of CAR T-cell therapy for MM, wherein long-term tumor control requires sustained immune activity within a dynamically evolving microenvironment.

### 5.4. Integrated View of Resistance

Collectively, MM resistance to CAR T-cell therapy arises from the convergence of antigen-related escape, intrinsic CAR T-cell dysfunction, and host- and microenvironment-mediated suppression. These multifactorial mechanisms indicate that durable therapeutic benefits cannot be achieved through advances in CAR construct design alone. Rather, the effective optimization of CAR T-cell therapy requires an integrated, biology-driven approach that addresses disease burden, antigen dynamics, CAR T-cell functional fitness, and the host immune context across the entire treatment continuum. In the following section, we discuss emerging strategies for optimizing CAR T-cell therapy by leveraging these biological insights to enhance efficacy, durability, and safety.

## 6. Strategies to Optimize CAR T-Cell Therapy for Multiple Myeloma

Optimizing CAR T-cell therapy for treating MM requires an integrated approach that addresses the multifactorial determinants of efficacy and mechanisms of resistance outlined in the preceding sections. Rather than relying solely on advances in CAR construct design, effective optimization strategies must encompass patient selection, treatment timing, pre-infusion disease control, and post-infusion management to maximize durable clinical benefits. These optimization strategies can be conceptualized within the integrated framework shown in Figure 1.

### 6.1. Patient Selection and CAR T-Cell Therapy Timing

Patient selection and treatment timing constitute critical variables that influence CAR T-cell outcomes. Accumulating evidence has suggested that the early use of CAR T-cell therapy (i.e., prior to the development of advanced disease burden and extensive T-cell dysfunction) may improve response durability [4,11,39]. Patients with preserved immune fitness, a lower tumor burden, and controlled disease kinetics are more likely to achieve a favorable E:T balance prior to infusion, thus supporting robust CAR T-cell expansion and persistence. These observations suggest that a strategic shift toward the earlier integration of CAR T-cell therapy within the MM treatment algorithm, rather than reserving it exclusively for late-line salvage settings, may prove beneficial [4,34].

### 6.2. Optimization of Pre-Infusion Disease Control

Pre-infusion disease control represents a central and actionable opportunity to optimize CAR T-cell therapy. As discussed in Section 3.1 and Section 3.2, tumor burden and responses to bridging therapy exert profound influences on post-infusion outcomes [2]. Effective bridging strategies should aim to achieve meaningful cytoreduction while preserving T-cell quality and minimizing lymphotoxicity. The rational selection of bridging regimens, tailored to disease biology and prior treatment exposure, may improve E:T ratios and mitigate early CAR T-cell exhaustion [34]. Notably, emerging clinical data suggest that the depth of response achieved during bridging therapy is directly associated with subsequent efficacy and safety outcomes following CAR T-cell infusion [4,39]. Bridging therapy, as an integral component of CAR T-cell treatment rather than a temporary logistical measure, is essential for improving the consistency and durability of responses.

### 6.3. Integration of Biological Markers into Clinical Decision-Making

Building on the biological determinants discussed in Section 3, recent advances have enabled the translation of these correlates into clinically actionable decision-making tools. Advances in the understanding of biological correlates of response have enabled the optimization of CAR T-cell therapy beyond CAR construct design alone. Key biomarkers, including the effector-to-target (E:T) ratio, antigen load, and sBCMA, provide actionable insights into the biological state of both tumor and CAR T-cell products at the time of infusion. Rather than maximizing CAR T-cell dose or peak expansion, accumulating evidence suggests that optimizing these biomarkers through effective tumor burden reduction prior to infusion may exert a disproportionate impact on post-infusion CAR T-cell functionality and durability. In this context, sBCMA serves as a practical and dynamic marker that can inform treatment timing, patient selection, and the effectiveness of bridging therapy. In parallel, emerging product-level functional assessments, such as quantitative evaluation of CAR molecule localization and clustering, offer complementary information regarding CAR T-cell fitness that is not captured by conventional tumor-based biomarkers [22]. Together, the integration of tumor burden reduction, dynamic biomarker assessment, and product-level functional characterization provides a rational framework for biologically informed clinical decision-making in CAR T-cell therapy for MM.

### 6.4. Combination and Sequential Strategies

Combination and sequential treatment strategies represent promising avenues for enhancing CAR T-cell efficacy and overcoming resistance [14,15]. These can include the use of alternative or dual-target CAR T-cell therapies following BCMA-directed treatment, integration with bispecific antibodies, or sequential immunotherapeutic strategies designed to sustain immune pressure in heterogeneous tumor populations [40]. Although such strategies introduce additional complexity, they also offer opportunities to overcome antigen escape and prolong disease control when applied in a biologically informed and patient-specific manner.

### 6.5. Toward a Design-Oriented and Patient-Centered Framework

The optimization of CAR T-cell therapy for MM ultimately requires a shift toward a design-oriented and patient-centered framework that integrates CAR engineering, disease biology, and immune-related factors of each patient [19]. Tailored CAR T-cell strategies may include biomarker-guided treatment timing, antigen-based target selection (e.g., BCMA versus GPRC5D), and rational sequencing of CAR T-cell therapy with other immunotherapeutic approaches based on disease biology and immune fitness. The continued integration of translational research with clinical practice will be essential to refining these strategies and improving the consistency and durability of CAR T-cell therapy for MM [34].

## 7. Future Perspectives

The future of CAR T-cell therapy for MM will likely be shaped by continued advances concerning both biological understanding and therapeutic design [19]. As insights into antigen dynamics, CAR T-cell functional fitness, and patient immune system contexts deepen, the field is moving toward increasingly precise and adaptable treatment strategies that extend beyond single-agent or single-target approaches.

One key direction involves the rational integration of next-generation CAR designs with biomarker-driven patient selection and treatment timing. Advances in dual-target and sequential immunotherapies, combined with the biologically informed optimization of pre-infusion disease control, are expected to mitigate resistance mechanisms such as antigen escape and T-cell dysfunction. In parallel, the incorporation of real-time biomarkers such as tumor burden metrics and product-level functional assessments may facilitate dynamic adjustments of therapeutic strategies across the treatment continuum. Beyond conventional CAR T-cell platforms, unconventional CAR-based approaches such as CAR-engineered natural killer T (CAR-NKT) cells have emerged as promising alternatives. CAR-NKT cells possess unique immunological properties that bridge innate and adaptive immune responses and mediate antitumor activity through CAR-dependent as well as endogenous T-cell receptor- and NK receptor-mediated mechanisms. Preclinical and early translational studies suggest that CAR-NKT cells may offer effective tumor control with favorable safety profiles, including reduced risks of graft-versus-host disease and cytokine release syndrome, warranting further clinical investigation in MM [41,42].

Another emerging strategy is in vivo CAR engineering, in which CAR constructs are delivered directly into patients to generate functional CAR-expressing immune cells without ex vivo manufacturing [43]. Early clinical experience in MM has demonstrated the feasibility of this approach and provided preliminary evidence of biological activity, including deep disease control such as minimal residual disease negativity [44]. Although this strategy remains at an early stage and faces challenges related to delivery specificity, safety, durability, and reproducibility, in vivo CAR engineering represents a potentially transformative platform that may complement existing CAR T-cell therapies in the future.

## Figures and Tables

**Table 1:** Clinically advanced BCMA-directed CAR T-cell therapies for multiple myeloma.

Product	Target	Key Trial	Patient Population	ORR	≥CR Rate	Median PFS	Key Toxicities	Notes
Idecabtagene vicleucel (ide-cel)	BCMA	KarMMa-3	RRMM (two to four prior regimens)	71%	39%	13.3 months	CRS, ICANS, cytopenia	First FDA-approved BCMA CAR-T; rapid responses but limited durability in high tumor burden
Ciltacabtagene autoleucel (cilta-cel)	BCMA	CARTITUDE-4	lenalidomide refractory MM	85%	73%	Not reached at a median follow-up if 15.9 months	CRS, ICANS, delayed neurotoxicity	

Abbreviations: BCMA, B-cell maturation antigen; CAR, chimeric antigen receptor; CRS, cytokine release syndrome; ICANS, immune effector cell-associated neurotoxicity syndrome; ORR, overall response rate; PFS, progression-free survival; RRMM, relapsed or refractory multiple myeloma. Cross-trial comparisons should be interpreted with caution due to differences in study design, patient populations, and follow-up durations.

## Data Availability

No new data were created or analyzed in this study. Data sharing is not applicable to this article.
